# Retrospective study revealed that Zn relate to improvement of swallowing function in the older adults

**DOI:** 10.1186/s12877-021-02224-8

**Published:** 2021-04-26

**Authors:** Yumika Seki, Kota Ishizawa, Tetsuya Akaishi, Michiaki Abe, Koji Okamoto, Junichi Tanaka, Ryutaro Arita, Shin Takayama, Akiko Kikuchi, Mariko Miyazaki, Hideo Harigae, Mayumi Sato, Masaya Hoshi, Kazuaki Hatsugai, Tadashi Ishii

**Affiliations:** 1 Department of Nephrology, Endocrinology and Vascular Medicine, Tohoku University Graduate School of Medicine, 1-1, Seiryo-machi, Aoba-ku, Sendai, 980-8574 Miyagi Japan; 2 Minamisanriku Hospital, 14-3, Numata Shizugawa Minamisanrikucho, Motoyoshigun, 986-0782 Miyagi Japan; 3 Department of Education and Support for Regional Medicine, Tohoku University Hospital, 1-1, Seiryo-machi, Aoba-ku, Sendai, Miyagi 980-8574 Japan; 4 Tohoku Medical Megabank Organization, Tohoku University, 2-1, Seryo-machi, Aoba-ku, Sendai, 980-8573 Miyagi Japan; 5 Department of Hematology and Rheumatology, Tohoku University Graduate School of Medicine, 1-1, Seiryo-machi, Aoba-ku, Sendai, 980-8574 Miyagi Japan; 6 Jikeien, Special Nursing Home for the Elderly, Social Welfare Corporation, 159-2, Doujishita, Iriyaaza, Minamisanrikucho, Motoyoshigun, 986-0782 Miyagi Japan

**Keywords:** Aspiration pneumonia, Dysphagia, Supplementation, Swallowing function, Zinc supplementation

## Abstract

**Background:**

Zinc is an essential micronutrient for maintaining biological activity. The level of zinc in the blood is known to decrease with age, especially in those over 75 years of age. In older adults patients with impaired functional status, aspiration pneumonia based on dysphagia often becomes problematic. However, the relationship between zinc deficiency and swallowing function has not been studied before.

**Methods:**

A total of 52 older adults subjects (15 males and 37 females) living in a nursing home were enrolled for this study. At the time of enrollment, data of gender, age, body weight, serum zinc levels, serum albumin levels, and the time in a simple 2-step swallowing provocation test (S-SPT) were collected. In patients with serum zinc levels < 60 μg/dL, we initiated 2 months of oral zinc supplementation therapy with a 34 mg/day zinc load. Those who underwent zinc supplementation were re-evaluated after the treatment period and serum zinc levels and S-SPT time were measured.

**Results:**

At the time of enrollment, serum zinc level was significantly correlated with serum albumin levels (Pearson’s R = 0.58, *p* < 0.0001) and time in the S-SPT (Spearman’s rho = − 0.32, *p* = 0.0219). Twenty-five of the 52 patients had zinc deficiency with a serum zinc level < 60 μg/dL. After 2 months of oral zinc supplementation, both serum zinc levels (*p* < 0.0001) and time in the S-SPT (*p* = 0.04) significantly improved. Meanwhile, serum albumin level (*p* = 0.48) or body weight (*p* = 0.07) did not significantly change following zinc supplementation. Zinc supplementation significantly improved swallowing function, especially in the older adults who had comorbid dysphagia and zinc deficiency.

**Conclusions:**

Zinc deficiency is associated with compromised swallowing function in older adults patients with impaired general functions. Oral zinc supplementation can alleviate dysphagia in older adults patients with zinc deficiency even though this is a retrospective study. Further study will be needed to confirm this positive effect.

**Supplementary Information:**

The online version contains supplementary material available at 10.1186/s12877-021-02224-8.

## Introduction

Zinc is an essential micronutrient vital for maintaining a variety of biological activities in living organisms. Zinc is among the building blocks of many enzymes, functional proteins, and living tissue cells [1, 2]. Zinc availability is known to decrease in the older adults, especially in those over 75 years of age [3]. Zinc deficiency causes various clinical symptoms, such as dysgeusia (i.e. distortion of taste), abnormal bone metabolism, skin lesions, gonadal dysfunction, decreased appetite, immune dysfunction [4, 5], and delayed wound healing [6, 7]. Studies show that zinc supplementation is effective in ameliorating these conditions, highlighting zinc’s homeostatic importance [8–17].

In the older adults, pneumonia-associated death is one of the leading causes of death worldwide [18]. Additionally, aspiration pneumonia is the most deliberating disease in the older adults resulting in impaired daily functional performance necessitating professional care. This condition is typically caused by aspiration of intraoral food debris during meals or saliva based on dysphagia [19] . There are many causes of dysphagia, and impaired throat sensation (i.e. laryngeal hypoesthesia) is one such cause [20]. Many symptoms arise from dysphagia. To date, there have been several reports indicating a possible relationship between zinc deficiency and dysphagia-associated pneumonia [21, 22], but there has been no report studying the relationship between zinc deficiency and laryngeal hypoesthesia. In this study, we proposed that zinc plays an important role in laryngeal sensation, apart from taste, and evaluated the relationship between zinc deficiency and laryngeal hypoesthesia.

## Material and methods

### Enrollment

This study was performed at a single public nursing home for the older adults in Japan. All older adults subjects who agreed to participate in this study and provided written informed consent were enrolled. Individuals who were found to not be mentally competent, due to dementia or other medical conditions, were enrolled only after a legal representative agreed to the patient’s participation and provided written informed consent. Those who did not agree to take part in this study or those whose legal representatives did not agree to participate were excluded.

### Studied variables

The following data were comprehensively collected at the initial enrollment for all patients: age, sex, past medical history, body weight, serum zinc level [μg/dL], serum albumin level [g/dL], and the required time to swallow using a simple 2-step swallowing provocation test (S-SPT). To account for circadian variation in serum zinc levels, all measurements of serum zinc were performed in the morning. Zinc was administered daily. S-SPT was performed only twice, before and after zinc administration for 2 months. The timing of S-SPT was done when the patient general condition was calm without fever, and the time of administration was in the afternoon when the patient was awake [23–25].

For patients who showed abnormally low serum zinc levels (i.e. ≤ 60 μg/dL) during enrollment, 150 mg/day of Polaprezinc (containing 116 mg L-carnosine and 34 mg zinc) was administered for 2 months [26]. In these patients, to assess the effects of zinc supplementation, follow-up measurement of serum zinc and albumin levels was performed at 2 months and the body weight was measured after 6 months from the enrollment.

### Measurement of S-SPT

S-SPT was performed according to the original report by Teramoto et al. [23]. The patients were laid flat on the bed during the test, but those who could not keep a flat position were tested in a 30–45-degree head-up position. After insertion of a 5-French children nasal catheter, approximately 13 cm from the nostril, the examiner investigated the mouth and confirmed that the tip of tube was correctly located in the oropharynx. Next, the examiner administered 0.4 cc of distilled water at room temperature through the inserted tube and measured the time taken to provoke a swallowing reflex. In cases where the swallowing reflex was not provoked after 30 s from the administration of 0.4 cc distilled water, an additional 2.0 cc of distilled, room-temperature water was administered. In order to normalize the time to swallow across all the patients, those who required the additional 2.0 cc of water to induce swallowing had 30 s added to their time, thus allowing for statistical convenience. S-SPT time in those who did not swallow, even with the additional 2.0 cc of distilled water, was regarded as 60 s. However, because this conversion was arbitrary, we selected non-parametric statistical methods when using the S-SPT data.

### Statistics

Comparisons of two unpaired variables were performed by the Student’s t-test or Mann-Whitney U test, based on the distribution patterns of the variables. Comparisons of two paired variables were performed by the paired t-test or Wilcoxon signed rank test, based on the distribution patterns of the variables. Comparisons of the prevalence between two or more unpaired groups were evaluated by a chi-squared test and those between two paired groups were evaluated by the McNemar’s exact test. For the estimation of correlation between two variables, Pearson correlation coefficient or Spearman’s rank correlation coefficient was calculated based on the distribution patterns of the two variables. Normality of distributions was evaluated with Kolmogorov-Smirnov test. *P*-values less than 0.05 were regarded as statistically significant. Statistical analyses were performed using JMP Pro 14 (SAS Institute Inc., Cary, NC, USA).

## Results

### Patient background and laboratory data

A total of 52 older adults patients (15 males and 37 females) were enrolled for this study. Clinical and laboratory data from the initial enrollment assessment for all 52 patients are summarized in Table 1. Twenty-five patients (48.1%) showed serum zinc level below 60 μg/dL, which is regarded as zinc deficiency. Patients were admitted into the nursing home for the following reasons: dementia in 37 patients (71.2%), cerebrovascular diseases in 13 patients (25.0%), and mental retardation in 2 patients (3.8%).

**Table 1:** Clinical and laboratory data of the initially enrolled 52 patients at the commencement of the study

Type of variable	Distributions (mean ± SD or median [IQR])
Male: Female	15: 37
Age	86.6 ± 7.2 years old
Body weight	46.2 ± 10.2 kg
PS 2	*n* = 3 (5.8%)
PS 3	*n* = 6 (11.5%)
PS 4	*n* = 43 (82.7%)
S-SRT ^a^	5.3 s [2.9–23.0 s]
Swallow with 0.4 cc	39 / 52 (75.0%)
Swallow with 2.0 cc ^b^	11 / 13 (84.6%)
Serum laboratory data	
Zinc level	59.3 ± 11.2 μg/dL
Albumin level	3.4 ± 0.4 g/dL
Total protein level	6.4 ± 0.6 g/dL
Total cholesterol	182.8 ± 34.1 mg/dL
White blood cell count	6072 ± 2143 /μL

*Abbreviations*: *IQR* interquartile range (25–75 percentile range), *PS* performance status, *SD* standard deviation, *S-SPT* simple 2-step swallow provocation test

^a^ S-SPT time in those who required 2.0 cc to swallow (*n* = 13) were converted by adding 30 s to their total time

^b^ Prevalence of swallow provocation with 2.0 cc of water was among those who did not swallow with 0.4 cc of water

### Correlation between serum biomarkers and S-SPT

**Fig. 1:**
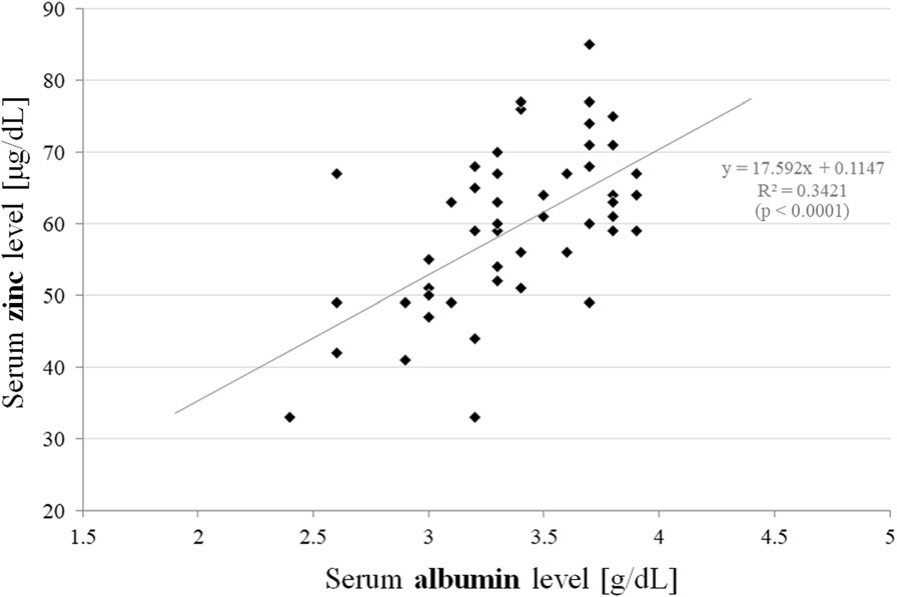
Correlation between serum zinc and albumin levels at time of enrollment

First, to evaluate the correlation between nutrition level and serum zinc level, we assessed the correlation between serum albumin and zinc level at enrollment. As a result, there was a strong positive correlation between these two variables with a Pearson’s R value of 0.58 (*p* < 0.0001, test of no correlation), as shown in Fig. 1.

**Fig. 2:**
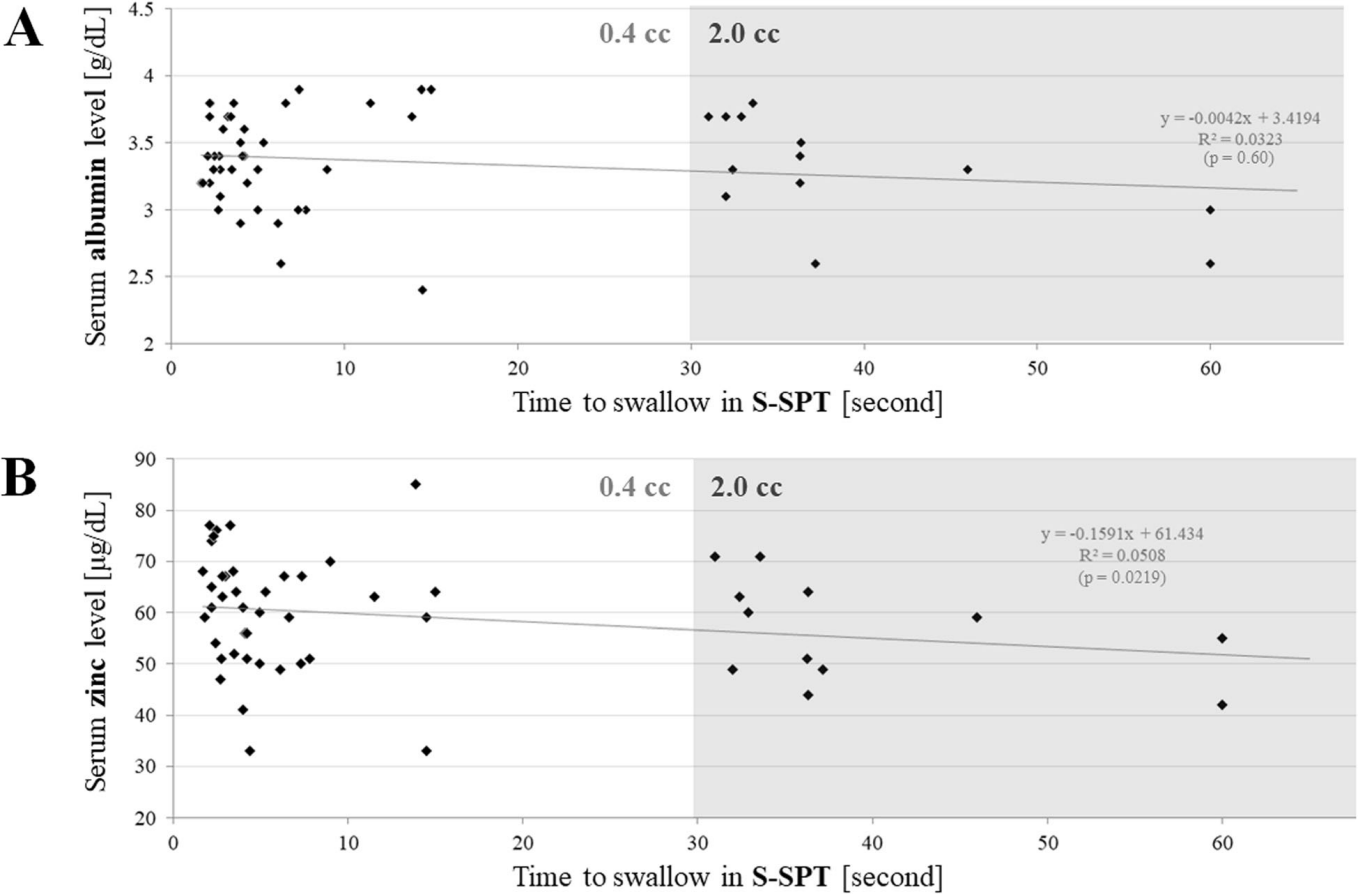
Correlation of serum albumin or zinc and S-SPT at time of enrollment. Abbreviation: S-SPT, simple 2-step swallowing provocation test. Shown *p*-values are the results of the test of no correlation with albumin, but significant correlation with zinc level. **a** Serum albumin level did not show a significant correlation with S-SPT time (Spearman’s rho = − 0.07; *p* = 0.60). **b** Serum zinc level showed a weak, but statistically significant, correlation with S-SPT time (Spearman’s rho = − 0.32; *p* = 0.0219)

Then, we assessed the correlation between the time taken in the S-SPT and each of these two serum biomarkers. As shown in Fig. 2a, serum albumin level did not show a significant correlation with S-SPT time (Spearman’s rho = − 0.07; *p* = 0.60). Meanwhile, as shown in Fig. 2b, serum zinc level showed a weak, but statistically significant, correlation with S-SPT time (Spearman’s rho = − 0.32; *p* = 0.0219).

### Effects of zinc supplementation to swallowing function

**Fig. 3:**
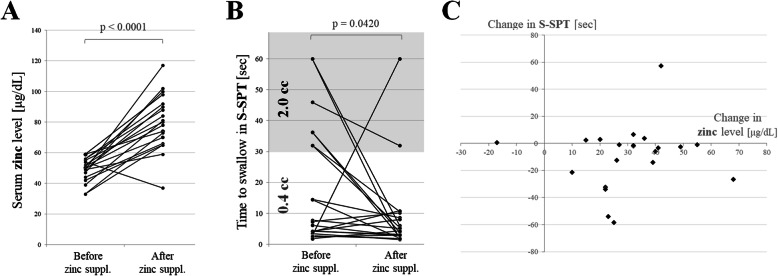
Changes in serum zinc and the S-SPT after oral zinc supplementation. Abbreviations: S-SPT, simple 2-step swallowing provocation test; suppl., supplementation. **a** The serum zinc level was significantly increased, with an initial mean and standard deviation of 49.4 ± 7.6 vs 79.9 ± 17.5 μg/dL post-treatment (*p* < 0.0001, paired t-test). **b** The S-SPT time was also significantly improved, with an initial median and interquartile range (IQR) of 7.32 [4.12–32.0] vs 4.28 [2.74–8.59] seconds post-treatment (*p* = 0.0420, Wilcoxon’s signed-rank test). **c** Although several patients showed both improved zinc levels and improved S-SPT time simultaneously, these two changes failed to show a statistical significance in combination (Spearman’s rho = 0.13; *p* = 0.576, test of no correlation)

Among the 25 patients whose serum zinc level at enrollment was < 60 μg/dL, 21 patients (84.0%), or their legal representatives, agreed to take zinc supplementation (i.e., 34 mg zinc load per day) for 2 months followed by another S-SPT. After the treatment period, as shown in Fig. 3a, the serum zinc level was significantly increased, with an initial mean and standard deviation of 49.4 ± 7.6 vs 79.9 ± 17.5 μg/dL post-treatment (*p* < 0.0001, paired t-test). As shown in Fig. 3b, the S-SPT time was also significantly improved, with an initial median and interquartile range (IQR) of 7.32 [4.12–32.0] vs 4.28 [2.74–8.59] seconds post-treatment (*p* = 0.0420, Wilcoxon’s signed-rank test). Meanwhile, serum albumin level (3.2 ± 0.3 vs 3.1 ± 0.5 g/dL; *p* = 0.48) or body weight (42.5 ± 7.0 vs 41.9 ± 7.2 kg; *p* = 0.0684) were not significantly changed after the zinc supplementation.

Based on the simultaneous improvement of serum zinc levels and the S-SPT time in the enrolled patients, we evaluated the correlation between the change in serum zinc level and the change in S-SPT resulting from zinc supplementation. As shown in Fig. 3c, although several patients showed both improved zinc levels and improved S-SPT time simultaneously, these two changes failed to show a statistical significance in combination (Spearman’s rho = 0.13; *p* = 0.576, test of no correlation).

## Discussion

In this study, we evaluated the serum zinc level in 52 older adults patients living in a nursing home. Previous research has shown that the serum zinc level in the general population decreases with age, as a result, many older adults subjects have a zinc deficiency. There are many possible explications for the observed decrease in zinc levels [4, 9, 17, 22, 27]. First, zinc intake from foods decreases with age and second, the elementary absorption of zinc from the intestinal tract decreases with age [3, 28, 29]. The third possibility is that, in those taking many medications, zinc is chelated by the drugs [30, 31]. Drugs with a chelating function bind to circulating zinc and are excreted in the urine; thus, it is less readily absorbed [32, 33]. Aside from chelating agents, drugs that increase the pH level in the gastroenteric tract, such as proton pump inhibitors, also decrease zinc absorption [30].

As in many previous reports, there was a positive correlation between serum zinc and albumin levels in the enrolled subjects. This was expected, because zinc is known to mostly bind to albumin in the blood [31]. Meanwhile, in this study, the serum albumin concentration was not affected by zinc supplementation. In addition, there was no significant correlation between the serum albumin level and the time in S-SPT, suggesting that the improvement in swallowing function depended solely on the improved zinc concentration, not on the serum albumin level or nutrition level. Zinc is involved in protein synthesis and enzyme activation; thus, after an adequate supplementation period, zinc supplementation eventually increase serum albumin level, BMI, and body weight. However, in this study, we could not confirm such changes in nutrition level-related markers after zinc supplementation, possibly because of the relatively short supplementation period of only 2 to 3 months. Future research should focus on the long-term effects of zinc supplementation in elderlies with zinc deficiency.

The serum zinc level in one patient, who did not take proton-pump inhibitor and other chelating agents, was not improved after zinc supplementation. The patient had a long history of having a bedridden status; although the reason is unclear, intestinal zinc absorption possibly have been compromised due to the already deteriorated health condition.

To explain the observed correlation between serum zinc levels and swallowing function, we hypothesized that zinc supplementation could have promoted functional improvement of swallowing-related neurons and increased the number of taste buds in the pharynx and larynx, leading to improved function of sensory input in the swallowing reflex pathway. In contrast to the taste buds in the tongue which exclusively function as gustatory receptors, those in pharyngeal and laryngeal regions are believed to also prevent pulmonary aspiration [31]. Additionally, several previous articles have reported that intracellular carbonic anhydrase activity is involved in the relationship between taste receptivity and zinc in the tongue [32, 33]. It is also suggested that carbonic anhydrase have some roles in the function of cells with these receptors and as well as neural transmission in the larynx.

Capsaicin is a molecule that is known to be associated with S-SPT and is believed to be useful in preventing aspiration pneumonia in the older adults [34]. Increased serum zinc level is suggested to increase capsaicin levels as well, thereby improving swallowing function. In this study, we did not check the levels of capsaicin; the further evaluation would be important to be able to clinically elucidate the correlation between zinc and capsaicin.

Cerebrovascular diseases are among the most common causes of dysphagia; damage in different areas within the central nervous system could promote dysphagia, such as delayed swallowing reflex, inadequate swallowing motion, and abnormal swallowing pattern. Impairment in swallowing reflex is not an only a consequence of dysphagia, and other multiple factors are often involved [35]. In this study, some of patients had brain vascular disease. In most of these patients, serum zinc levels were not improved after oral zinc supplementation, and the swallowing function was only minimally affected. Meanwhile, those whose serum zinc levels were severely decreased without the history of cerebrovascular diseases responded better to the oral zinc supplementation both in their serum zinc level and in their swallowing functions (supplement Figure 1). Future research in the older adults with zinc deficiencies should better stratify their treatment population to more clearly identify factors that distinguish responders and non-responders to oral zinc supplementation.

## Conclusions

Zinc deficiency was associated with impaired swallowing function based on laryngeal hypoesthesia in the older adults with impaired general functional status. Oral zinc supplementation successfully recovered the swallowing function as seen by improvement in swallowing provocation time. Oral zinc supplementation is a promising therapeutic strategy to alleviate dysphagia in older adults who show both zinc deficiency and prolonged swallowing provocation time. Although this was a retrospective study, further prospective research with a placebo control will be needed to confirm this positive effect.

## Supplementary Information

**Additional file 1.**

**Additional file 2.**

## Data Availability

The full dataset generated and analyzed during the current study are not publicly available in order to maintain the privacy of the patients during this study. De-identified data can be made available from the corresponding author on reasonable request.

## Abbreviations

S-SPT: Simple 2-step swallowing provocation test
ACE2: Angiotensin-converting enzyme 2
COVID-19: Coronavirus Disease 2019
IQR: interquartile range (25–75 percentile range)
PS: performance status
SD: standard deviation
