# Supplementary material for: Retrospective study revealed that Zn relate to improvement of swallowing function in the older adults
Source: BMC Geriatr. 2021 Apr 26;21:279. doi: 10.1186/s12877-021-02224-8 (PMC8075009; doi:10.1186/s12877-021-02224-8)
Supplement: Supplementary file 1 — Additional file 1. [file 12877_2021_2224_MOESM1_ESM.docx]

Supplement Figure 1

After Zn supplementation S-SPT with brain infarction improved from 19.1sec to 2.8sec P=0.0312 wilcoxon matched-pairs; whereas group without brain infarction improved from 6.2sec to 5.1sec P=0.30 wilcoxon matched-pairs.

Supplement Figure 2

95% CI = 20.59 ~ 37.11

supplement figure 3

95% CI = -20.33 ~ 1.593
