# Supplementary material for: Retrospective study revealed that Zn relate to improvement of swallowing function in the older adults
Source: BMC Geriatr. 2021 Apr 26;21:279. doi: 10.1186/s12877-021-02224-8 (PMC8075009; doi:10.1186/s12877-021-02224-8)
Supplement: Supplementary file 2 — Additional file 2. [file 12877_2021_2224_MOESM2_ESM.pdf]

ID	MMSE	PS	Gender	S-SPT	Alb	TP	LYP	Ht	WBC	Hb	TG	Cho	BUN	CRE	Zn	Disease	Age	PPI	TC	RA drug	levodopa	urosemid	CE inhibito	NSAID	Body Weight	Zn (B)	TC (B)	Alb (B)	S-SPT (B)	BMI (B)	BW (B)	Zn (A)	TC (A)	Alb (A)	S-SPT (A)	BMI (A)	BW (A)
1	0	4	F	36.3	3.2	6.1	1183	34.1	6800	11.7	48	148	6.9	0.35	44	AD	84	-	-	-	-	+	-	-	35.8	44	148	3.2	36.3	17.7	38.6	66	162	2.6	3.89	15.9	34.5
2	0	4	F	3.48	3.3	6.3	1386	38.6	10500	12.6	271	194	13.4	0.46	52	AD	85	-	-	-	-	+	-	-	39	52	194	3.3	3.48	21.2	38.2	84	128	2.4	1.57	20.9	37.6
3	2	3	F	4.23	3.4	5.8	1348	37.4	4200	12.6	311	265	18.3	0.74	51	AD	83	-	-	-	-	-	-	-	51.5	51	265	3.4	4.23	25.9	53	78	285	3.7	3.17	25.8	52.8
4	1	4	M	60	3	5.9	528	36.4	4400	12.2	51	129	11.2	0.77	55	AD	86	-	-	-	-	-	-	-	49.7	55	129	3	60	16.1	47.3	80	133	2.9	1.6	18.5	47.3
5	10	4	M	6.15	2.9	5.7	2059	35.5	7200	11.6	98	144	25	0.88	49	AD, CD	91	-	-	-	-	+	-	-	37.3	49	144	2.9	6.15	17.3	39.9	90	140	2.9	2.74	17.6	40.7
6	3	4	M	14.47	3.9	6.8	975.6	30.9	3600	10.6	49	133	11.6	0.72	59	mental retardation	75	-	-	-	-	-	+	+	41.5	59	133	3.9	14.47	17.6	42.9	85	132	4	2	17.6	42.8
7	2	4	F	36.29	3.4	6.1	1459	34	5700	11.6	142	176	9.9	0.53	51	CD	84	-	-	-	-	-	-	-	41.5	51	176	3.4	36.29	19.5	42.4	73	140	3.2	2.43	20.2	43.8
8	11	4	M	2.78	3.4	6.1	1872	36.6	5200	12.6	105	243	6.9	0.66	39	AD	93	+	-	-	-	-	-	-	66.8	39	243	2.8	2.78	23	64.1	81	197	3	60	22.3	62.5
9	2	4	F	4.12	3.4	6.5	1283	43	4900	13.9	111	224	11.6	0.44	56	AD	94	+	-	-	-	-	-	-	47.2	56	224	3.4	4.12	25	45.9	88	228	3.3	10.78	24.9	45.7
10	0	4	F	2.72	3	5.9	841.5	32.7	3300	10.6	86	169	17.8	0.59	47	CD	96	-	-	-	-	-	-	-	34.8	47	169	3	2.72	20.3	37.8	102	182	3.2	1.85	20.3	37.7
11	13	4	M	60	2.6	5.1	1368	35.5	6000	12	45	194	13.5	0.96	42	CD	89	-	-	-	-	-	-	-	44.4	42	194	2.6	60	19.7	43.7	65	187	2.3	6.07	18.3	40.7
12	14	4	F	4.37	3.2	5.9	474.5	38.8	6500	13.3	63	169	14.1	0.7	33	CD	75	+	-	-	-	-	+	-	30.8	33	169	3.2	4.37	13.7	31.6	65	139	3.5	2.88	13.5	31.1
13	1	4	M	4.23	3.6	7	1200	34.5	4800	11.4	73	188	10.2	0.72	56	AD	88	-	-	-	-	-	-	-	44.7	56	188	3.6	4.23	16.8	45.6	92	175	3.4	8.07	16.5	44.7
14	10	4	M	4.98	3	6.2	1095	36.3	7400	12.4	90	139	17.1	0.69	50	deafness	98	-	-	-	-	-	-	-	53.2	50	139	3	4.98	NA	53.2	49	109	2.6	NA	NA	NA
15	4	4	M	2.43	3.3	7.2	1652	32	6800	10.4	89	146	19.6	1.21	54	AD	93	-	-	-	-	-	-	-	42.5	54	146	3.3	2.43	17.6	43.4	37	86	2.4	3.03	15.6	38.6
16	6	4	F	7.8	3	6.3	2956	37.6	7600	12.5	231	220	18.3	0.57	51	AD	85	-	-	-	-	-	+	-	54.7	51	220	3	7.8	23.8	52.1	100	233	3.1	5.14	23.4	51.3
17	0	4	F	45.98	3.3	6.6	1710	25.2	3600	8.2	108	144	26.9	1.22	59	AD	88	-	-	-	-	-	-	-	39.7	59	144	3.3	45.98	16.1	36.3	98	136	3.6	31.96	16	35.9
18	10	4	M	32	3.7	7	1474	35.7	5400	12.2	79	175	11.6	0.72	49	AD, CD	69	-	-	-	-	-	-	+	50.3	49	175	3.7	32	19.5	50.9	59	166	3.2	10.58	19.7	51.1
19	10	4	F	14.5	2.4	5.5	1949	24.4	6700	7.7	124	130	12.5	0.52	33	AD	93	-	-	-	-	-	-	-	55.3	33	130	2.4	14.5	24.2	55.9	73	122	2.3	8.59	24.2	56
20	9	4	F	32	3.1	6	1586	36.4	5200	11.7	123	202	13.5	0.87	49	AD	96	-	-	-	-	-	-	-	34.7	49	202	3.1	32	16.1	36.2	117	223	3.4	5.45	16.1	36.3
21	0	4	F	1.84	3.2	6.4	NA	36.3	4500	11.9	116	206	19.1	0.48	59	AD, brain hemorrhage	85	-	-	-	-	-	-	-	29.7	59	206	3.2	1.84	13.7	29.7	74	221	3.5	4.28	13.7	29.6
22	0	4	F	7.32	3	5.9	NA	30.1	6500	9.5	104	163	19.1	0.89	50	AD	84	+	-	-	-	-	-	-	39.1	50	163	3	7.32	21.1	39.1	70	176	3.8	10.19	21.1	39
																		4	0	0	0	3	3	2													
																		18.2%	0.0%	0.0%	0.0%	13.6%	13.6%	9.1%													
23	14	2	F	3.99	3.5	6.2	1148	38.4	4300	12.8	158	169	13.5	0.54	61	AD	94	-	-	-	-	-	-	-	43.1												
24	21	3	F	13.9	3.7	7	1787	42.1	10900	14.3	128	232	17.8	0.71	85	AD, Congenital hip joint Disease	84	-	-	-	-	+	-	-	38.5												
25	9	4	F	36.35	3.5	5.9	1529	41.6	6400	13.5	77	187	22.1	0.45	64	Femoral head and neck fracture	98	-	-	-	-	-	-	-	36.1												
26	0	4	F	3	3.6	5.8	939.6	30.5	5400	12.5	129	236	13.2	0.79	67	AD	84	-	-	-	-	-	-	-	37.8												
27	0	4	F	2.22	3.7	7.3	1885	38.5	5000	12.9	249	182	10	0.72	74	AD, CD	90	-	-	-	-	-	-	-	45.7												
28	0	4	F	5.32	3.5	7	3229	40.1	8800	13	127	169	8.1	0.63	64	AD, CD	82	-	-	-	-	-	+	-	41												
29	4	4	F	2.84	3.1	5.2	579.6	40	6900	13.9	56	125	20	0.67	63	lewy body disease	92	-	-	-	-	-	-	-	40.2												
30	0	4	F	6.34	2.6	5.5	1632	30.7	5300	9.4	83	132	11	0.51	67	AD	95	-	-	-	-	-	-	-	43.3												
31	0	4	F	2.82	3.3	6.7	619.4	42.1	3800	13.9	99	171	5.5	0.7	67	AD, CD	82	-	-	-	-	-	+	-	57.6												
32	1	4	M	15	3.9	6.8	123	33.5	4100	10.8	84	166	13.2	0.65	64	AD	90	-	-	-	-	-	-	+	46.8												
33	9	3	F	3.3	3.7	6.5	2180	34	7900	11.3	97	143	11.7	0.9	77	AD	87	+	-	-	-	-	+	-	55												
34	17	3	F	2.5	3.4	5.7	1915	38.3	5700	12.1	119	161	18.3	0.75	76	AD	94	+	-	-	-	+	-	+	55.6												
35	5	4	M	3.62	3.8	7.4	NA	37	6300	12	202	227	25.3	1.3	64	AD	85	-	-	-	-	-	-	-	58												
36	24	2	F	6.62	3.8	6.6	2053	30.9	6800	9.4	87	205	38.5	1.67	59	AD	92	-	-	-	-	-	-	+	61.5												
37	15	3	M	2.3	3.8	7.3	1237	43.3	4600	14.7	161	187	23.8	1.78	75	CD	82	+	-	-	-	-	-	-	66												
38	1	4	F	3.46	3.7	6.7	2738	39.2	7400	13.3	248	227	15.5	0.73	68	AD	75	-	-	-	-	-	-	-	56.6												
39	20	3	F	31.02	3.7	7.1	3547	39.4	8100	13	113	181	9.5	0.5	71	depression	78	-	-	-	-	-	-	-	47												
40	0	4	F	4.98	3.3	7.2	939.9	36.7	3900	11.4	124	201	23.8	0.57	60	AD	91	-	-	-	-	+	-	-	31.4												
41	1	4	F	1.7	3.2	5.8	1191	40.9	7400	13.3	161	197	17.5	0.53	68	AD	79	-	-	-	+	-	-	-	33.5												
42	1	4	F	11.5	3.8	7.4	860.4	40.8	3600	13.6	105	183	12.5	0.68	63	AD, SAH, CD	81	-	-	-	-	-	-	+	39.1												
43	14	2	M	7.39	3.9	6.7	824	43	4000	14.5	75	179	11.7	0.83	67	CD	73	-	-	-	-	-	-	-	59.6												
44	1	4	F	2.23	3.2	7	2044	41.4	7600	12.9	61	189	12.6	0.65	65	AD	94	-	-	-	-	-	-	-	37.5												
45	0	4	F	32.42	3.3	7.4	1580	37.7	7600	12.6	102	233	18.5	1.32	63	AD	74	-	-	-	-	+	-	-	76												
46	4	4	M	8.99	3.3	7.6	1262	35.9	6100	11.2	54	198	14.3	0.59	70	AD	86	+	-	-	-	-	-	+	53.4												
47	1	4	F	33.56	3.8	6.3	1705	37.3	4900	12.3	117	210	15.4	0.54	71	AD, PD	79	-	-	-	+	-	-	-	32.9												
48	23	4	F	2.09	3.4	6.2	2227	35.8	5500	12	461	214	16.9	0.81	77	AD	84	-	-	-	-	-	-	+	50.8												
49	15	4	F	32.92	3.7	6.5	1120	38.8	4500	12.6	166	196	19.8	0.78	60	CD	89	-	-	-	-	-	-	-	50.1												
50	0	4	F	2.2	3.8	6.4	1052	42.9	2900	13.6	87	209	12.7	0.44	61	mental retardation	84	-	-	-	-	-	-	-	39												
51	0	4	F	4.03	2.9	6.1	1247	35.2	14500	12.3	59	142	24.6	0.56	41	AD	88	-	-	-	-	-	-	-	35.4												
52	5	4	M	37.17	2.6	6.1	1338	38.5	7600	13.3	84	156	12.3	0.53	49	AD	99	-	-	-	-	+	-	-	NA												
																		4	0	0	2	5	3	6													
																		13.8%	0.0%	0.0%	6.9%	17.2%	10.3%	20.7%	45.739216												

MMSE	Mini Mental State Examination
PS	Performance Status
PPI	Prot Pomp inhibitor
TC	Tetracycline hydrochloride
RA	Rumatoid Arthritis
ACE	Angiotensin-Converting Enzyme
NSAID	Non-Steroidal Anti-Inflammatory Drug
S-SPT	simple 2-step swallow provocation test
PD	Parkinson Disease
AD	Alzheimer Disease
SAH	Subarachnoid Hemorrhage
CD	Cerebrovascular Disease
